# Volumetric mass density measurements of mesenchymal stem cells in suspension using a density meter

**DOI:** 10.1016/j.isci.2022.105796

**Published:** 2022-12-10

**Authors:** Christoph Drobek, Juliane Meyer, Robert Mau, Anne Wolff, Kirsten Peters, Hermann Seitz

**Affiliations:** 1 Chair of Microfluidics, University of Rostock, 18059 Rostock, Germany; 2 Department of Cell Biology, Rostock University Medical Center, 18057 Rostock, Germany; 3 Department of Life, Light and Matter, University of Rostock, 18059 Rostock, Germany

**Keywords:** Stem cells research, Biophysics, Biology experimental methods

## Abstract

To use regeneratively active cells for cell therapeutic applications, the cells must be isolated from their resident tissues. Different isolation procedures subject these cells to varying degrees of mechanical strain, which can affect the yield of cell number and viability. Knowledge of cell volumetric mass density is important for experimental and numerical optimization of these procedures. Although methods for measuring cell volumetric mass density already exist, they either consume much time and cell material or require a special setup. Therefore, we developed a user-friendly method that is based on the use of readily available instrumentation. The newly developed method is predicated on the linear relationship between the volumetric mass density of the cell suspension and the volumetric mass density, number, and diameter of the cells in the suspension. We used this method to determine the volumetric mass density of mesenchymal stem cells (MSCs) and compared it to results from the established density centrifugation.

## Highlights

• Method allows determination of volumetric mass density of cells in suspension
• Method is user-friendly and based on readily available instrumentation
• Method is time-saving and requires fewer cells
• Method has been used to determine the volumetric mass density of MSC

## Graphical abstract

**Figure:**
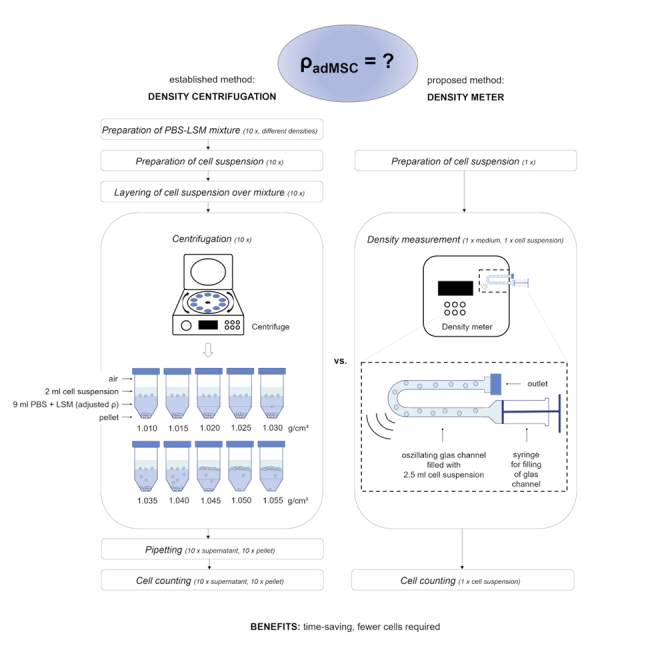

## Introduction

Mesenchymal stem/stromal cells (MSCs) are increasingly used in cell therapeutic applications because of their regenerative potential.^1^^,^^2^ To enable clinical use, the cells can be isolated from their resident tissues (e.g., adipose tissue or bone marrow) by a broad variety of technical procedures, which can be of manual or automated nature.^3^^,^^4^ The cell isolation procedures include processes such as agitation, filtration, and centrifugation that put mechanical strain on the cells. The cell media used to investigate the resistance of cells to mechanical shear and strain in an experimental setting need to be adjusted for the volumetric mass density of the cells.^5^ Furthermore, the technical procedures mentioned above can be investigated using computational fluid dynamics (CFD) simulations to gain additional insights and reduce development time and costs compared to trial-and-error experiments. They allow the investigation of mechanical strain and mechanical forces and can predict the trajectory of individual cells in cell suspensions using particle models based on the Euler-Lagrange approach.^6^^,^^7^^,^^8^ These models also require the volumetric mass density of the cells as an input parameter. Lastly, Grover et al.^9^ report changes in cell volumetric mass density during biological processes such as cell cycle progression, apoptosis, differentiation, and certain diseases.

Differences between cell volumetric mass densities were originally utilized for the separation of different cell types via density gradient centrifugation, e.g., for the separation of different blood cell types (reviewed in Pertoft^10^). Since then, multiple liquid media have been established for this technique;^11^ among them are Ficoll^12^ and Percoll.^13^ Density gradient centrifugation has also been used to evaluate the cell density of human cells,^14^ freshwater phytoplankters,^15^ and polymer nanoparticles^16^ quantitatively. This technique is material and time consuming, and due to the multiple manual steps that must be performed, it is prone to human error.

Suspended microchannel resonators (SMRs) have also been used in recent years for the determination of mass and volumetric mass density of biomolecules.^17^^,^^18^ They facilitate the measurement of the volumetric mass density of individual cells, particles, or many single cells in succession^9^^,^^19^^,^^20^^,^^21^ using yeast cells, transfused or malaria-infected human erythrocytes, human lung cancer cells, and mouse lymphoblasts. The SMR’s working principle is similar to that of the density meter used in this study, only downscaled to the micrometer range. It consists of an oscillating microcantilever with a microchannel through which fluid and cells are flown. A single cell in the fluid changes the mass and resonance frequency of the oscillating cantilever. The volumetric mass density can then be calculated from these changes. However, since the microcantilever works under a vacuum for increased signal quality, the original manufacturing process of the measuring equipment is complex, but advances in the fabrication of SMRs have been reported.^22^ Still, the technology is patented and currently only commercially available for particle or cell diameters below 5 μm (Malvern Panalytical). Also, optically induced electrokinetics has been used in a microfluidic chip to lift single cells and track the sedimentation via microscope and camera.^23^ This technique also has an easier fabrication process than SMRs. In other experiments, glass or silica capillaries have been stretched to decrease their diameter, measure the resonance frequency, and thereby determine the buoyant mass.^24^^,^^25^ Another approach is measuring the drag force of a single cell in a microfluidic channel on a lab-on-chip microfluidics system together with a syringe pump and an inverted microscope.^26^ The study has been performed using yeast cells. However, there are currently no commercially available systems using all these approaches either.

Because the available techniques for determining the volumetric mass density of cells are either complex and time consuming or not widely commercially available, we established a user-friendly method for these cells using commercially available hardware such as density meters and cell counters. Both devices are already available in many laboratories.

Within this study, we refer to this method as cell suspension density measurement since it is key to measuring the volumetric mass density of the cell suspension which is increased by cells with higher volumetric mass density. In addition to measuring the volumetric mass density of the cell suspension, the volumetric mass density of the suspension medium, as well as the number of cells and the average cell diameter, has to be measured as accurately as possible. To evaluate its validity, we compared this novel method to the classic approach of measuring cell volumetric mass density via density centrifugation. For this study, we used primary human MSC from adipose tissue (adMSC).

### Cell suspension density measurement: Theoretical approach

Because the volumetric mass density of the cell suspension and the suspension medium can be measured via density meter and the number of cells in the cell suspension and their average diameter can be measured via cell counter, the average volumetric mass density of the suspended cells can be calculated as follows:

The mass of a cell suspension $m_{susp}$ is added up from the mass of the medium $m_{medium}$ (continuous phase) and the mass of the dispersed phase $m_{cells}$ (Equation 1).

(Equation 1) $$m_{susp}=m_{medium}+m_{cells\;,}$$

where

(Equation 2) $$m_{susp}=\rho_{susp}{\;V}_{susp\;,}$$

(Equation 3) $$m_{medium}=\rho_{medium}{\;V}_{medium\;,}$$

(Equation 4) $$m_{cells}=\rho_{cells}{\;V}_{cells}$$

Being able to measure the volumetric mass densities $\rho_{susp}$ and $\rho_{medium}$ and specify the volumes $V_{susp}$, $V_{medium}$, and $V_{cells}$, one can calculate the volumetric mass density of the cells

(Equation 5) $$\rho_{cells}=\frac{\rho_{susp}V_{susp}-\rho_{medium}V_{medium}}{V_{cells}}$$

that are dispersed in the continuous fluid phase.

While $V_{susp}$ is the sample volume for the density measurement, the total volume of all cells in the sample of the cell suspension (total cellular volume)

(Equation 6) $$V_{cells}=n_{cells}\;\frac{1}{6}\;\pi \;d_{cells,avg}^{3}$$

is calculated from the cell number $n_{cells}$ and the volume of a single cell, modeled here as perfect sphere with diameter

(Equation 7) $$d_{cells,avg}=\sqrt[{3}]{\frac{6}{\pi \sum n_{d}}\sum_{d_{\min}}^{d_{\max}}\frac{1}{6}\pi d^{3}n_{d}}$$

The diameter $d_{cells,avg}$ is volume-averaged and calculated from the cell diameter distribution with the number of cells $n_{d}$ at a diameter d in a range from $d_{\min}$ to $d_{\max}$. The medium volume

(Equation 8) $$V_{medium}=V_{susp}-V_{cells}$$

is then simply the difference between suspension volume and total cell volume.

## Results

Cell suspension density measurement was used to determine the volumetric mass density of cultured human adMSC isolated from the tissue of 6 different donors. For comparison, the volumetric mass density of the same cell material was measured using the conventional method of density centrifugation.

### Cell suspension density measurement: Determination of the cell volumetric mass density using a density meter

**Figure 1:**
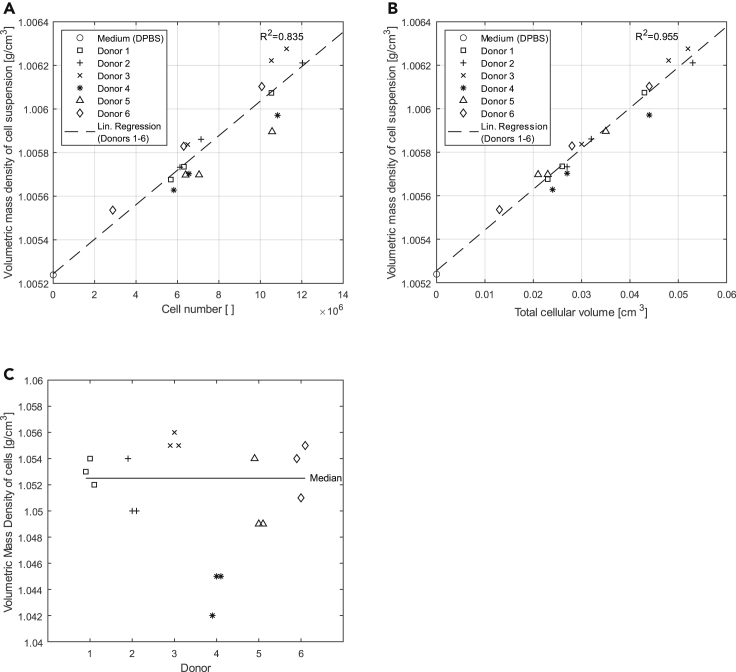
Cell suspension density measurement Volumetric mass density of cell suspension (n = 6) and DPBS plotted (A) against the cell number and (B) against the total cellular volume for donors 1–6. R^2^ of the linear regression (dashed line) increased by plotting against the total cellular volume. The total cellular volume accounts not only for the cell number but also for the diameter of the cells. The better regression underlined the importance of the proper calculation of the average diameter (using a volume average) from the diameter distribution. (C) Volumetric mass density of the cells (n = 6). The fluctuations in the individual cell samples 1–3 for each donor are by factor 3 smaller than the fluctuation over all cell samples of all six donors. It is also noteworthy that the determined cell volumetric mass densities of donor 4 are 0.004 g/cm^3^ lower than the lowest determined volumetric mass density of the other five donors.

The calculated volumetric mass density of the adMSC in the cell suspension had a median of 1.0525 g/cm^3^ (Table 1, Figure 1C). It was not normally distributed and varied between 1.042 and 1.056 g/cm^3^ which was less than 1% fluctuation from the median. While the range was 0.014 g/cm^3^ over all measurements of all six donors, it was slightly smaller with 0.001 g/cm^3^ to 0.005 g/cm^3^ for an individual donor. Note that donor 4 had marginally lower cell volumetric mass densities than the other donors.

**Table 1:** Determined cell volumetric mass density (column 8) of adMSC derived from Equation 5 as well as the inputs for Equation 5 such as sample volume (column 3), cell number (column 4), volume-averaged cell diameter (column 5), and the measured volumetric mass density of the cell suspension (column 6) and the medium (column 7). For each donor, three individual cell suspensions (cell samples 1-1 to 6-3) with different cell numbers and therefore concentrations were investigated. The results for the cell samples 1-1 to 6-3 are listed as arithmetic averages of the three technical replicates that were performed per cell sample

1	2	3	4	5	6	7	8
Donor	Cell sample	Sample volume of cell suspension for density meter	Number of cells (total) in cell suspension sample	Volume-averaged diameter of cells in suspension	Volumetric mass density of cell suspension	Volumetric mass density of the medium (DPBS)	Volumetric mass density of cells in cell suspension via Equation 5
		$V_{susp}$ [ml]	$n_{cells}$ []	$d_{cells,avg}$ [μm]	$\rho_{susp}$ [g/cm^3^]	$\rho_{medium}$ [g/cm^3^]	$\rho_{cells}$ [g/cm^3^]
1	1-1	2.5	6,310,000	19.83	1.005735	1.005239	1.053
	1-2	2.5	10,533 333	19.83	1.006074	1.005239	1.054
	1-3	2.5	5,677 000	19.83	1.005676	1.005239	1.052
2	2-1	2.5	7,136 666	20.39	1.005861	1.005247	1.054
	2-2	2.5	12,033 333	20.39	1.006211	1.005247	1.050
	2-3	2.5	6,120 000	20.39	1.005733	1.005247	1.050
3	3-1	2.5	6,493 333	20.60	1.005837	1.005251	1.055
	3-2	2.5	10,533 333	20.60	1.006222	1.005251	1.056
	3-3	2.5	11,266 666	20.60	1.006276	1.005251	1.055
4	4-1	2.5	5,820 000	19.79	1.005628	1.005278	1.042
	4-2	2.5	10,833 333	19.79	1.005971	1.005278	1.045
	4-3	2.5	6,553 333	19.79	1.005703	1.005278	1.045
5	5-1	2.5	6,391 666	18.47	1.005697	1.005290	1.054
	5-2	2.5	10,566 666	18.47	1.005896	1.005290	1.049
	5-3	2.5	7,041 666	18.47	1.005698	1.005290	1.049
6	6-1	2.5	6,300 000	20.31	1.005829	1.005287	1.054
	6-2	2.5	10,070 000	20.31	1.006103	1.005287	1.051
	6-3	2.5	2,875 000	20.31	1.005536	1.005287	1.055
Median							1.0525

The determined adMSC volumetric mass density (Table 1, column 8) was 5% higher than that of the used medium (Dulbecco’s PBS [DPBS]). The adMSC did increase the volumetric mass density of the cell suspension (Table 1, column 6) by up to 0.001 g/cm^3^ or 0.083% for the performed measurements, compared to the volumetric mass density of the medium. The deviation from the median is less than 1% for all 6 donors.

The volumetric mass density of the cell suspension was therefore dependent on the cell number and, according to Equation 6, also on the total cellular volume in the cell suspension. It increased linearly with cell number (with R^2^ of the linear regression being 0.835) and also with the total cellular volume $V_{cells}$ (R^2^ of the linear regression 0.955). The linear fits not only matched well with the underlying data but also matched with the volumetric mass density of the DPBS solution (Figure 1A at zero cell number, Figure 1B at zero total cellular volume).

### Determination of the cell volumetric mass density using density centrifugation

The results in this section were determined using the commonly practiced density centrifugation and were intended to validate the volumetric mass density of adMSC that was obtained by using the proposed cell suspension density measurement.

**Figure 2:**
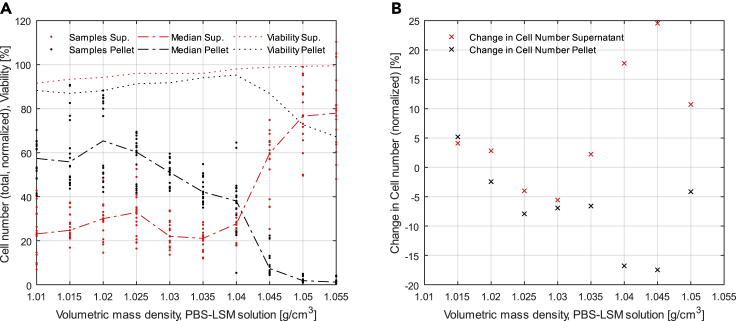
Density centrifugation (A) Total number of cells after centrifugation normalized to total number of cells before centrifugation (n = 6). Shown are the technical replicates of all individual cell samples (black dots) and their median (dashed black line) for the cell pellet and the supernatant (red dots and dashed red line) as well as the average viability of the cells in the pellet (dotted black line) and in the supernatant (dotted red line). (B) Change in the normalized cell number for pellet (black) and supernatant (red) for n = 6. The peak of change occurs both for pellet and supernatant at a volumetric mass density of 1.045 g/cm^3^.

The results in this section are presented as normalized cell numbers that are present in the different DPBS-LSM solutions with their varying density after the centrifugation. The DPBS-LSM solutions are mixtures of DPBS and Lonza Lymphocyte Separation Medium (LSM) (Lonza Group AG, Switzerland). In Figure 2A, the normalized total number of cells is plotted over the volumetric mass density of the different DPBS-LSM mixtures. All cell numbers are shown normalized, meaning relative to the number of cells initially filled into the 2 mL cell suspension before centrifugation. With an increased volumetric mass density of the DPBS-LSM mixture, fewer cells sedimented into the pellet during the centrifugation because the forces leading to sedimentation become smaller with decreasing density difference between the adMSC and DPBS-LSM mixture.

From 1.040 to 1.045 g/cm^3^, the cell number in the pellet decreased rapidly, while at the same time the cell number in the supernatant increased. At this DPBS-LSM volumetric mass density, cells were not able to sink through the DPBS-LSM mixture anymore. At DPBS-LSM volumetric mass densities higher than 1.045 g/cm^3^, the normalized total cell number in the pellet was close to 0% while the normalized total cell number in the supernatant was close to 100%. The magnitude of change in the normalized total cell number at different DPBS-LSM mixture densities indicates the actual volumetric mass density of the adMSC. Therefore, the change of the normalized total cell numbers is calculated using Equations 10 and 11 and plotted over the density of the DPBS-LSM solution (Figure 2B). Figure 2B clearly shows where the change in cell number peaks both for pellet and supernatant. The positive and negative peaks for supernatant and pellet near 1.045 g/cm³ indicate that the cells stopped transferring through the DPBS-LSM mixture and their volumetric mass density therefore should match the volumetric mass density of the DPBS-LSM solution. The raw data used to draw Figures 2A and 2B are included in Table S1.

**Figure 3:**
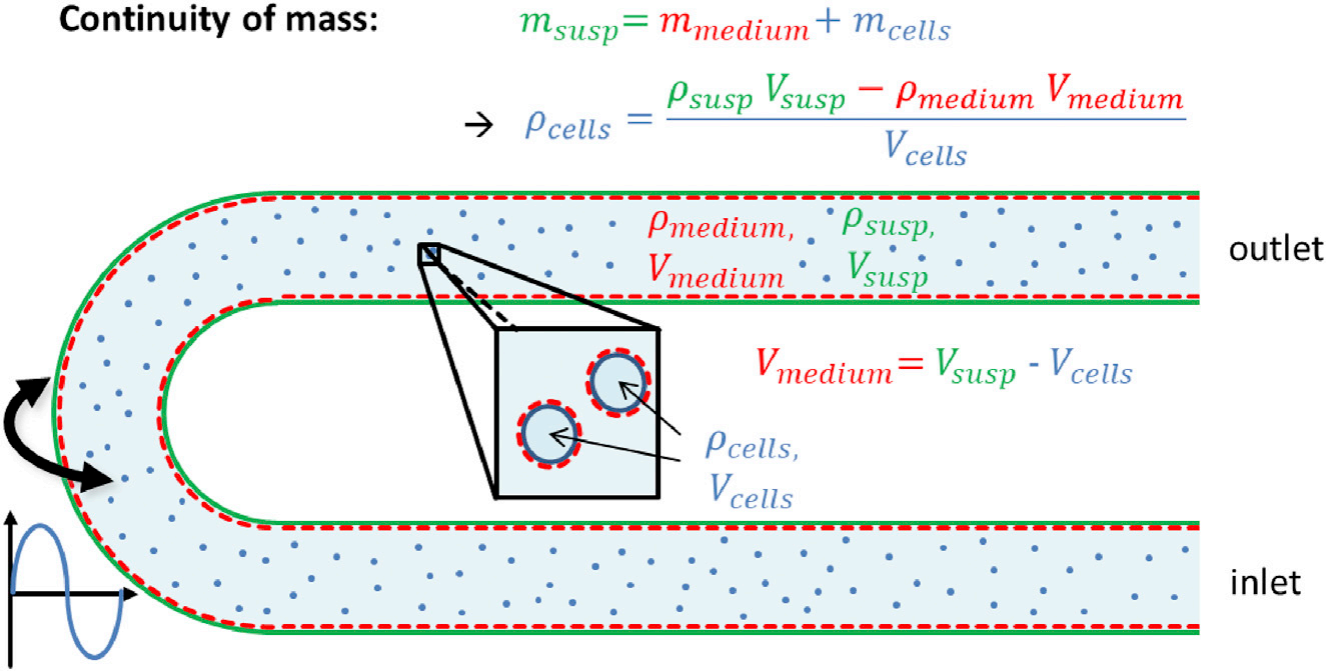
Measurement principle of DSA 5000 M density meter (Anton Paar GmbH, Austria) The resonance frequency of the oscillation of the glass tube is dependent on the density of the fluid in the glass tube. For a cell suspension, it is a mixture of a continuous fluid (the cell suspension medium) and a dispersed phase (the cells) that displaces the medium.^5^

**Figure 4:**
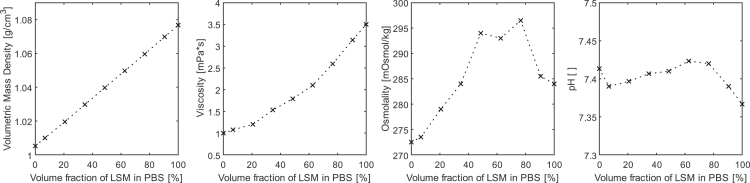
Volumetric mass density, dynamic viscosity, osmolality, and pH of different DPBS-LSM mixtures The exact fluid properties are listed in Table S2.

**Figure 5:**
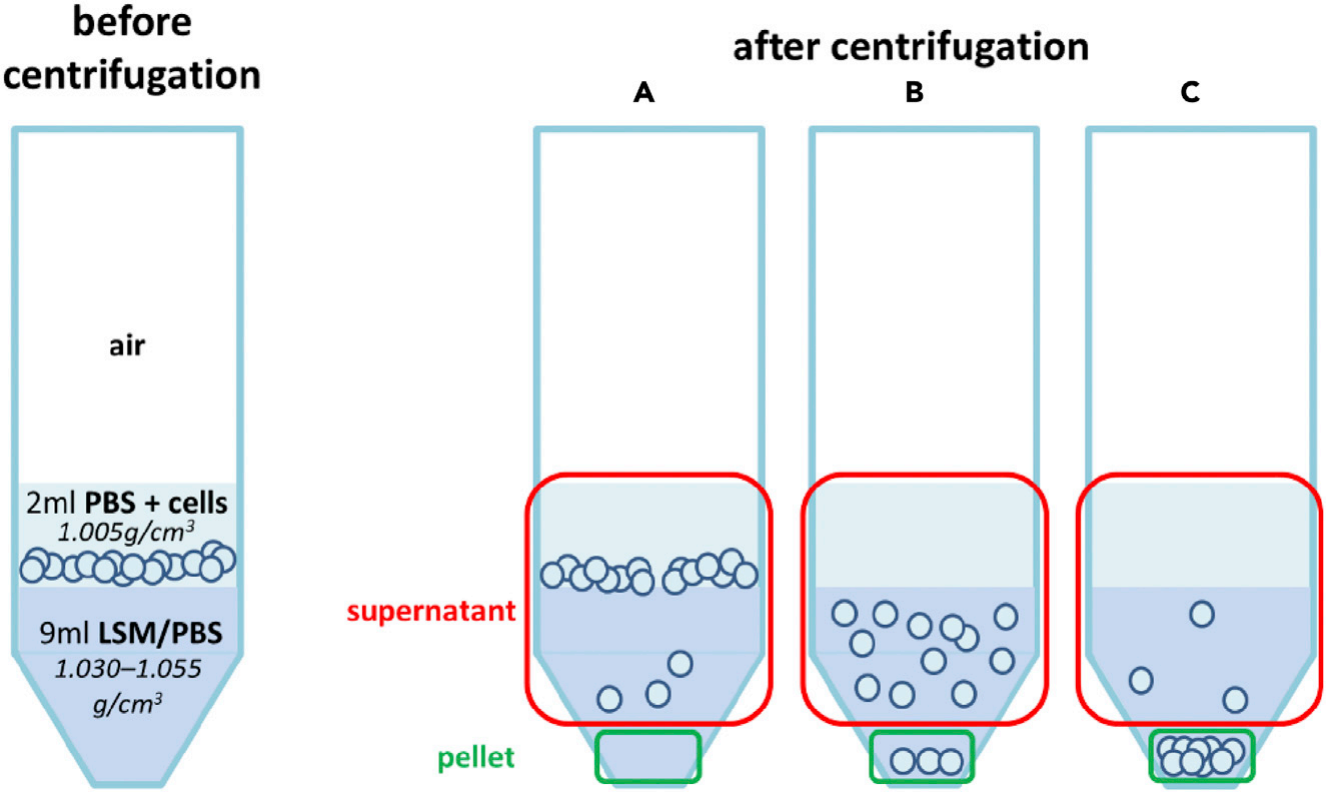
Concept of density centrifugation With the volumetric mass density of the cells being higher than the volumetric mass density of the DPBS (1.0052 g/cm^3^) that carries the cells, they always sink to the interface of DPBS and DPBS-LSM during centrifugation. (A) If the cell’s volumetric mass density is lower than the volumetric mass density of the respective DPBS-LSM mixture, the cells will remain at this interface. (B) If the volumetric mass density of the cells matches the volumetric mass density of the DPBS-LSM mixture, the cells will disperse throughout the DPBS-LSM mixture nearly homogeneously. (C) If the cells have even a higher volumetric mass density than the DPBS-LSM mixture, they will transfer through the DPBS-LSM and sink to form a pellet at the bottom of the centrifugation tube.

The methodology used for cell suspension density measurement (Figure 3) and density centrifugation (Figures 4 and 5) is described in the STAR Methods section of this paper.

## Discussion

### Evaluation of the results

In this study, we determined the volumetric mass density of adMSC by measuring the volumetric mass density of cell suspension and cell medium with a density meter. We refer to this procedure as cell suspension density measurement. For comparison, we also performed density centrifugation measurements (a simplified version of the well-established density gradient centrifugation) on the same patient’s material. We determined a median adMSC volumetric mass density of 1.0525 g/cm^3^ using our method and 1.045 g/cm^3^ (both for pellet and supernatant) using density centrifugation. We interpret this slight difference in volumetric mass density as the fact that nonviable cells and cell debris with their higher density^27^^,^^28^ are not considered in the calculation of cell volumetric mass density using the proposed method but are still present in the actual cell suspension. Therefore, nonviable cells and cell debris increase the measured volumetric mass density of the cell suspension and thus also increase the calculated cell volumetric mass density. During density centrifugation, cell debris simply sinks into the pellet and is not counted by the NC-200 cell counter.

We are not aware of any previously published measurements of the adMSC volumetric mass density. However, the volumetric mass density of related cell or tissue material has been determined previously. For fibroblasts, a volumetric mass density of 1.03 g/cm^3^ to 1.05 g/cm^3^ has been determined.^29^^,^^30^ For skeletal muscle tissue, a volumetric mass density of 1.06 g/cm^3^ has been measured.^31^^,^^32^ For fat cells (adipocytes) that differentiate from adMSC but contain a lipid vacuole, a volumetric mass density of 0.92 g/cm^3^ has been determined.^33^ The above-mentioned volumetric mass densities of fibroblasts were determined using density centrifugation while the volumetric mass density of skeletal muscle and adipose tissue was simply calculated from their dimensions and weight.

For the comparison of our new method for measuring the cell suspension density, we chose the standard density gradient centrifugation in a simplified version as it does not require a completely new setup, unlike SMR and drag force. Because there is no gradient of volumetric mass density in the solution, the cells can only be in two different locations in the tube after centrifugation: in the pellet, if the cells have a higher volumetric mass density than the DPBS-LSM solution used or in the supernatant (DPBS solution + DPBS-LSM solution), if the cells have a lower or equal volumetric mass density. This approach significantly reduces the required precision, and thus reduces the susceptibility to errors in multistage pipetting procedures, as is common with standard density gradient centrifugation and the subsequent necessary processing steps. Density centrifugation has further limitations: because it only allows the determination of the cell volumetric mass density by determining the (maximum) change of cell number for DPBS-LSM solutions with different densities, it only provides approximate results. It is therefore less precise than the newly presented method, which is clearly reflected in the more differentiated results of cell suspension density measurements (Figure 1C) compared to density centrifugation (Figure 2B). Furthermore, the resolution depends on the step size of the mixed DPBS-LSM solutions. At the same time, it takes much longer to perform the density and cell number measurements for every individual DPBS-LSM solution. Therefore, substantially more cellular material is needed, thus limiting the number of measurements that can be performed. It does not take into account cell debris and therefore shows slightly lower cell volumetric mass densities compared to the volumetric mass density measurements of cell suspension and medium. It is ultimately also based on a density meter and a cell counter and thus reliant on the same hardware as our proposed method of cell suspension density measurement.

On the other hand, the resolution of the volumetric mass density measurements of cell suspension and medium depends on the accuracy of the density meter and the cell counter and not on the method itself. Therefore, both the accuracy of the proposed method and its sensitivity are discussed here.

### Accuracy of the proposed method

The DSA 5000 M density meter has an accuracy^34^ of 0.000007 g/cm^3^ and a repeatability of 0.000001 g/cm^3^. The NC-200 cell counter has a theoretical coefficient of variation (CV) of 2.7% at a cell concentration of 1 × 10^6^ cells/mL compared to CVs of more than 10% for manual counting in hemocytometers.^35^

Preparation and handling of the cell suspension samples are crucial for the proposed method, as is the accuracy of the measurement systems. The exact number of cells in the sample and the exact volume of the cell suspension sample $V_{susp}$ are essential for the calculation of the cell volumetric mass density $\rho_{cells}$ via Equation 5. The cell suspension sample is therefore prepared using microliter pipettes with high accuracy. The accuracy of the scale (0.1 mL) of the 2.0 mL inlet syringe is insignificant for its subsequent filling with the cell suspension sample since the cell suspension is not completely loaded into the density meter and a part of the cell suspension sample remains in the syringe. It is important to do the measurements with the density meter directly after preparing the cell suspension sample. Otherwise, the cells will sediment and aggregate which may affect the density measurement. Air bubbles that can impair the density measurements are easily visible on the monitor of the DSA 5000 M density meter and can therefore be removed from the measurement glass tube with the help of the 0.5 mL extra volume that has been filled into the syringe.

The volumetric mass density of the cell suspension increases linearly with the number of cells in the sample. The high coefficient of determination of the regression (R^2^ = 0.835) confirms this hypothesis and suggests only small fluctuations between the measurements. Calculating the total cellular volume from cell number and volume-averaged diameter increases the R^2^ of the regression even further (R^2^ = 0.955). It is also noteworthy that the measured volumetric mass density of the DPBS medium has not been included in the regression but is touched by the regression line for a cell number or total cellular volume of zero. This shows the accuracy of the proposed method. Also, it confirms the accuracy of the density meter and cell counter which is (together with the precise handling of the samples) essential for an exact calculation of the cell volumetric mass density. For all six individuals, the volumetric mass density fluctuates only at the third decimal place while the first two decimal places are stable (Table 1). This demonstrates that our method even allows us to distinguish between individuals. Also, the good agreement with the results from density centrifugation using cells from the same patients and finally the good agreement with the volumetric mass densities of similar cell and tissue material from the literature indicate that the proposed method can accurately and reproducibly determine the volumetric mass density of a given cell type.

### Sensitivity of the proposed method

For the adMSC volumetric mass density, distinct clusters are formed from the measurement of the individual cell samples for every donor. The highest determined cell volumetric mass density belonging to donor 4 is about 0.004 g/cm^3^ lower than the lowest determined cell volumetric mass density of the other five donors. Simultaneously, the cell volumetric mass densities determined from the three individual cell samples of donor 4 vary by only about 0.003 g/cm^3^, so this variation is less than the difference from the other five donors. It is also noteworthy that the cell volumetric mass densities based on the individual cell samples of the other five donors fluctuate between 0.001 and 0.005 g/cm^3^ with donor 4 lying exactly in the middle of this range. Because the maximum donor-individual fluctuation of cell volumetric mass density (0.005 g/cm^3^ for donor 5) is also smaller than the fluctuation of cell volumetric mass density across all six donors (0.014 g/cm^3^) by about a factor of 3, there is a clear indication that different donors might have slightly different individual adMSC volumetric mass densities and that this distinct and donor-individual adMSC volumetric mass density can be determined by the proposed method.

With this method, it is possible to adjust the volumetric mass density of cell media, e.g., to investigate the resistance of cells to mechanical shear and strain or to perform numerical flow simulations. This is consistent with Grover et al.^9^ who show that there are slight differences in volumetric mass density for other cell types such as erythrocytes or L1210 mouse lymphocytic leukemia cells. Nevertheless, it remains a challenge to distinguish these donor-specific differences in cell volumetric mass density from measurement inaccuracies. However, with the precautions mentioned at the beginning of this section regarding the handling of the cell suspension and cleaning of the measurement glass tube before use (using 99.7% ethanol), it is possible to reduce the fluctuations of the measurements significantly enough to determine these donor-specific differences. Increasing the number of donors as well as the number of cell samples per donor could also increase the accuracy of the determinations of the volumetric mass density of a certain cell type and the donor-specific differences. Although three technical replicates per cell sample seem to be sufficient, this number could be increased to improve accuracy. With our proposed method, it could be further investigated as to whether there is a correlation between the difference in cell volumetric mass density and other cellular properties or pathologies, as outlined by Grover et al.^9^

In the context of density centrifugation, it is noteworthy that the viability of the few cells that passed through the DPBS-LSM solutions with densities higher than 1.040 g/cm^3^ and ended up in the pellet decreased by about 30%. We believe that this effect is due to the increased viscosity and resulting higher shear forces of the DPBS-LSM solution at higher volumetric mass densities. This phenomenon could underline the fact that the volumetric mass density and viscosity of cell media are important parameters for procedures where cell suspensions are handled, especially if they experience high shear. It consequently underlines the need for density-adjusted cell media.

We have developed a new method for determining the volumetric mass density of cells in suspension. This method is based on measuring the volumetric mass density of a cell suspension and its pure cell medium with a density meter and counting the cells in the suspension and then calculating the volumetric mass density of the cells in the cell suspension. We tested this new method with human adMSC and found that the volumetric mass density determined was consistent with the volumetric mass density of the same patient material determined by density centrifugation. Thus, the method we developed shows more precise results, since the measurement resolution depends only on the precision of the density meter and not on the number of mixed solutions (DPBS-LSM) or even a gradient. In addition, the new method requires less time and substantially fewer cells.

### Limitations of the study

This study presents an approach to determine volumetric mass density of cells in suspension. However, there are some limitations to this study. This study has a small sample size, but this specific study is only an illustration of a use case and the results for the volumetric mass density are close to each other. Also, only female donors were available for this study. It would be interesting to also include male donors in future studies to see if the volumetric mass density of cells is dependent on gender.

## STAR★Methods

### Key resources table

**Table:** 

REAGENT or RESOURCE	SOURCE	IDENTIFIER
**Biological samples**		

Human mesenchymal stem/stromal cells	Rostock University Medical Center	Ethics committee of Rostock University Medical Center, registration number: A2013-0112

**Chemicals, peptides, and recombinant proteins**		

0.25% Trypsin-EDTA, 1x	Thermo Fisher Scientific	Ref. 25,200-056
10% Fetal Calf Serum (FCS), South Africva Origin, Health class 1a, sterile filtered	PAN-Biotech	Cat# P30-1506
Dulbecco’s Modified Eagle Medium (DMEM) + GlutaMax-I + 4.5 g/L D-Glucose + Pyruvate, 1x	Thermo Fisher Scientific	Ref. 31,966-021
Dulbecco’s PBS (DPBS), w/o Calcium, w/o Magnesium, sterile filtered	PAN-Biotech	Cat# P04-36500
Penicillin(10,000 Units/ml)/Streptomycin(10,000 μg/mL)	Thermo Fisher Scientific	Ref. 15,140-122

**Software and algorithms**		

FlowJo v10	Flowjo	https://www.flowjo.com
MATLAB	Mathworks	https://www.mathworks.com
NucleoView NC-200	ChemoMetec	https://chemometec.com/nucleoview-software/
NucleoView NC-3000	ChemoMetec	https://chemometec.com/nucleoview-software/

**Other**		

Cell Culture Flasks, 250 mL, 75 cm^2^, PS, sterile	Greiner Bio-One	Cat# 658175
Centrifuge Tube, 50 mL, PP, graduated, conical bottom, sterile	Greiner Bio-One	Cat# 227261
Dynabeads CD34 Positive Isolation Kit	Thermo Fisher Scientific	Ref. 11301D
Via1-Cassette	ChemoMetec	Cat# 941-0012

### Resource availability

#### Lead contact

Further information and requests for resources and reagents should be directed to and will be fulfilled by the lead contact, Christoph Drobek (christoph.drobek@uni-rostock.de).

#### Materials availability

This study did not generate new unique reagents.

### Experimental Model and subject details

The patient material for this study was obtained with the patient’s informed consent. The study was approved by the ethics committee of Rostock University Medical Center [http://www.ethik.med.uni-rostock.de/], registration number A2013-0112 and it complies with the ethical standards defined by the World Medical Association Declaration of Helsinki. It concerns the adMSC of 6 female donors with the age range of 24–49 years. The median age is 41.5 years, the mean is 39.33 years.

The isolation of the cells from human adipose tissue has been previously described.^36^ After isolation, the cells were cultured in tissue culture flasks (Greiner Bio-One GmbH, Austria). The cell culture medium consisted of Dulbecco’s Modified Eagle Medium (DMEM, Thermo Fisher Scientific Inc., USA) supplemented with 1% penicillin/streptomycin (P/S, Thermo Fisher Scientific Inc., USA) and 10% fetal calf serum (FCS, PAN-Biotech GmbH, Germany).

After 24 h of adherence, cells positive for the surface marker CD34 were separated from other plastic-adherent cells using a magnetic bead selection system (Thermo Fisher Scientific Inc., USA). The cells thus obtained were further cultivated in tissue culture flasks. When they reached confluence after about 5 days, the cells were detached from the flask with 0.25% trypsin-EDTA (Thermo Fisher Scientific Inc., USA). They were divided in a 1:3 surface ratio onto new tissue culture flasks. This step was repeated after another 5 days. When the cells on the resulting flasks had then reached confluence, they were used for the two methods of cell volumetric mass density determination. The cells were detached from the culturing flasks with 0.25% trypsin-EDTA and were transferred into Dulbecco’s PBS (DPBS, PAN-Biotech GmbH, Germany) with 10% FCS. After centrifugation (5 min, 400 x g, room temperature), the cells were transferred into culture medium. The few potentially remaining magnetic beads from the CD34 selection were removed with a magnet. The adMSC were then stored on ice for further use.

### Method details

#### Cells suspension density measurement: Density meter

Prior to the measurements in the DSA 5000 M density meter (Anton Paar GmbH, Austria) the cell number and cell viability in the cell suspension were determined with the NucleoCounter NC-200 cell counter (ChemoMetec A/S, Denmark). The cell suspension was then divided into three fractions containing three different cell concentrations suitable for providing a reliable signal in the density meter. The range of suitable cell concentrations (‘cell samples’) had been determined in preliminary testing. Each of the three fractions were centrifuged (5 min, 400 x g, room temperature) and suspended in 3 mL DPBS. Of those 3 mL, 500 μL were used for another measurement of the cell number, cell viability, and cell diameter distribution with the NC-200. All three parameters were measured in technical triplicates of the same sample. The results of these measurements are displayed as average values of the technical triplicate measurements. The remaining 2.5 mL of the suspension were used for the measurement of the volumetric mass density in the DSA 5000 M density meter. In addition to the volumetric mass density of the cell suspension $\rho_{susp}$ the volumetric mass density of the suspension medium (DPBS), $\rho_{medium}$, was measured using the density meter.

The DSA 5000 M contains an oscillating U-shaped borosilicate glass tube filled with the fluid or suspension (Figures 3 and S1A). The resonance frequency of the oscillation of the U-shaped glass tube and therefore its period $T_{U}$ is dependent on the volumetric mass density of the sample

Equation (9) $$\rho_{sample}=A\frac{T_{U}}{T_{ref}}f_{1}-B{*f}_{2}$$

$T_{U}$ and $T_{ref}$ are the period of the U-shaped glass tube and the period of a reference oscillation while A and B are adjustment constants. $f_{1}$ and $f_{2}$ are correction functions for temperature and viscosity.^34^

A 2.0 mL syringe (B. Braun SE, Germany) was filled with $V_{susp}=2.5\;ml$ and was connected to the inlet of the density meter via a standard Luer adapter. The plunger was removed from a 1.0 mL syringe (B. Braun SE, Germany) and the barrel was then connected to the outlet of the density meter via a standard Luer adapter and served as a capillary. This setup allowed for reducing the sample volume to 2.5 mL but leaving room to pull or push out possible air bubbles (Figure S1B) during the filling of the system. The sample was cooled down to the target temperature of 20.0°C via the built-in Peltier element. This took about 5 min per sample. To avoid sedimentation and aggregation of cells the measurements had to be undertaken as quickly as possible. To avoid cellular residues in the measurement chamber that may affect the following measurements, after each measurement the glass tube was cleaned with distilled water and undenatured ethanol >99.7%. Lastly, the glass tube was dried with an air stream.

Both cell number and cell diameter distribution were measured with the NC-200 and the Via1 cassette (both ChemoMetec A/S, Denmark). The cell number, the standard output of this device, was displayed as a normalized cell count per mL. From this measured value, $n_{cells}$ was then calculated for the density measurement sample volume of 2.5 mL.

Because the average diameter of the NC-200 standard output was calculated as an arithmetic average, it was not consistent with the total cellular volume and their specific diameter. Since the cell diameter distribution could be extracted from the NC-200 raw data, the volume-averaged cell diameter was exported and calculated. It must be noted that the diameter distribution from the NC-200 was not exported as in cells/ml but in actual cell events from the measurement window. Therefore, in Equation 7 $\sum n_{d}$ the sum of all cells of all diameters in the cell counting sample was not equal to $n_{cells}$ which comes also from the cell counter but is projected to the 2.5 mL density measurement sample.

The NC-200 dataset was loaded into the NC-3000 software (ChemoMetec A/S, Denmark) where the dataset was exported in the fluorescence-activated cell sorting (FACS) data format. The data were then loaded into the FACS analysis software FlowJo v10 (Flowjo, LLC, USA) and the diameter distribution was exported from the histogram using the Windows Clipboard (Microsoft Corporation, USA). The diameter distribution could then be imported and the volume-averaged cell diameter $d_{cells,avg}$ could be calculated in any spreadsheet software, e.g., Excel (Microsoft Corporation, USA) via Equation 7. For adMSC diameters between $d_{\min}=1\;\mu m$ and $d_{\max}=41\;\mu m$ were included in the calculation of the volume-averaged cell diameter.

#### Cell volumetric mass density: Density centrifugation

To validate the results from cell suspension density measurements, a second method was performed: density centrifugation.

After measuring the cell number of the cell suspension (see section experimental model and subject details) using the NC-200 a portion of the cell suspension containing 20 x 10^6^ cells was centrifuged (5 min, 400 x g, room temperature) and suspended in 21 mL DPBS. 500 μL were used for cell number and cell diameter measurement with the NC-200 and 20 mL of the suspension were divided into 2 mL aliquots placed over different media with different volumetric mass densities to determine the cell’s volumetric mass density via density centrifugation. These media were mixtures of DPBS and Lonza Lymphocyte Separation Medium (LSM) (Lonza Group AG, Switzerland). LSM itself is a mixture of sodium diatrizoate (Hypaque) and Ficoll and has a density of 1.077 g/cm^3^ while DPBS has a density of 1.0052 g/cm^3^. DPBS-LSM mixtures with the volumetric mass densities of 1.010, 1.015, 1.020, 1.025, 1.030, 1.035, 1.040, 1.045, 1.050 and 1.055 g/cm^3^ were prepared.

After the preparation of the DPBS-LSM mixtures, the volumetric mass densities of the DPBS-LSM mixtures were verified using the DSA 5000 M. In addition, the dynamic viscosities, osmolalities, and the pH of the DPBS-LSM mixtures were determined to ensure cell-friendly conditions such as an osmolality in the isosmotic range between 275 and 290 mOsmol/kg and a pH around 7 over the entire mixture range from 100% DPBS to 100% LSM. The dynamic viscosity was measured using a Haake MARS II rotational rheometer (Thermo Fisher Scientific Inc., USA), the osmolality using an Osmomat 3000 (Gonotec GmbH, Germany), and the pH using a Seven Compact (Mettler Toledo Inc., USA). Both volumetric mass density and dynamic viscosity increased with increasing volume fraction of LSM in the DPBS-LSM solution. The fluid properties of the DPBS-LSM mixture are illustrated in Figure 4.

The 2 mL of the cell suspension were carefully layered over 9 mL of DPBS-LSM mixture with one of the 10 defined volumetric mass densities (Figure 5, left) in a 50 mL centrifugation tube (Greiner Bio-One GmbH, Austria). The tube was centrifuged for 5 min at 400 x g at room temperature. The cell numbers in the complete supernatant (consisting of DPBS and the DPBS-LSM mixture with parts of the cells) and in the pellet were determined using the NucleoCounter NC-200.

To interpret the density centrifugation data, the change (first derivative) of the normalized total cell number in the pellet (blue) and the supernatant (red) was plotted against the volumetric mass density of the DPBS-LSM mixture. The change in normalized total cell number at a given DPBS-LSM volumetric mass density was calculated from both neighboring points using a central differencing scheme as in Equations 10 and 11.

(Equation 10) $$f^{\prime }\left(\rho \right)=\frac{f\left(\rho +h\right)-f\left(\rho -h\right)}{2h}$$

(Equation 11) $$\Delta n=\frac{f\left(\rho +h\right)-f\left(\rho -h\right)}{2\Delta \rho }$$

### Quantification and statistical analysis

The details for all statistical analyses are provided in the table and figure legends. The statistical analyses were performed using MATLAB (The Mathworks Inc., USA). n = 6 represents the number of donors used in this research, for both cell suspension density measurement using the density meter as well as for density centrifugation.Performing cell suspension density measurement, triplicate samples from each donor were used. Cell counting was also done with technical triplicates for each donor sample. The technical replicates were averaged using the arithmetic mean. The cell diameters have been volume-averaged from the cell counter’s diameter distribution according to Equation 7 for every technical replicate and then the arithmetic mean of all 3 technical replicates of all 3 samples from a donor has been calculated. The median volumetric mass density of all 3 replicates of all 6 donors is used to represent the adMSC volumetric mass density determined from the cell suspension density measurement. Variances of the median have been judged in terms of % deviation. Variances between samples of a donor and variances between donors have also been compared in terms of the range. To further judge the validity of the theoretical approach as well as the accuracy of the measurements, linear regressions have been performed for the relationship between the measured cell suspension volumetric mass density and both cell number as well as total cellular volume using MATLAB. The coefficient of determination R^2^ has been computed for both regressions. For density centrifugation, cell counting of the supernatants and pellets of all six donors was done in technical triplicates. The technical replicates were averaged using the arithmetic mean. The median of the normalized cell numbers of all technical replicates for all 6 donors was calculated for both supernatant and pellet of every DPBS-LSM mixture with respective density. The adMSC volumetric mass density was then derived from the maximum change of normalized cell number using a central differencing scheme from the median of both supernatant and pellet.

## Data Availability

• This paper does not report original code. • Any additional information required to reanalyze the data reported in this paper is available from the lead contact upon request.

## Supplemental information

Document S1.Figure S1 and Table S2

Table S1. Raw data for the determination of the cell volumetric mass using density centrifugation, related to Figure 2
