# Supplementary material for: Volumetric mass density measurements of mesenchymal stem cells in suspension using a density meter
Source: iScience. 2022 Dec 10;26(1):105796. doi: 10.1016/j.isci.2022.105796 (PMC9803822; doi:10.1016/j.isci.2022.105796)
Supplement: Document S1.Figure S1 and Table S2 [file mmc1.pdf]

iScience, Volume *26*

Supplemental information

Volumetric mass density measurements of mesenchymal stem cells in suspension using a density meter

Christoph Drobek, Juliane Meyer, Robert Mau, Anne Wolff, Kirsten Peters, and Hermann Seitz

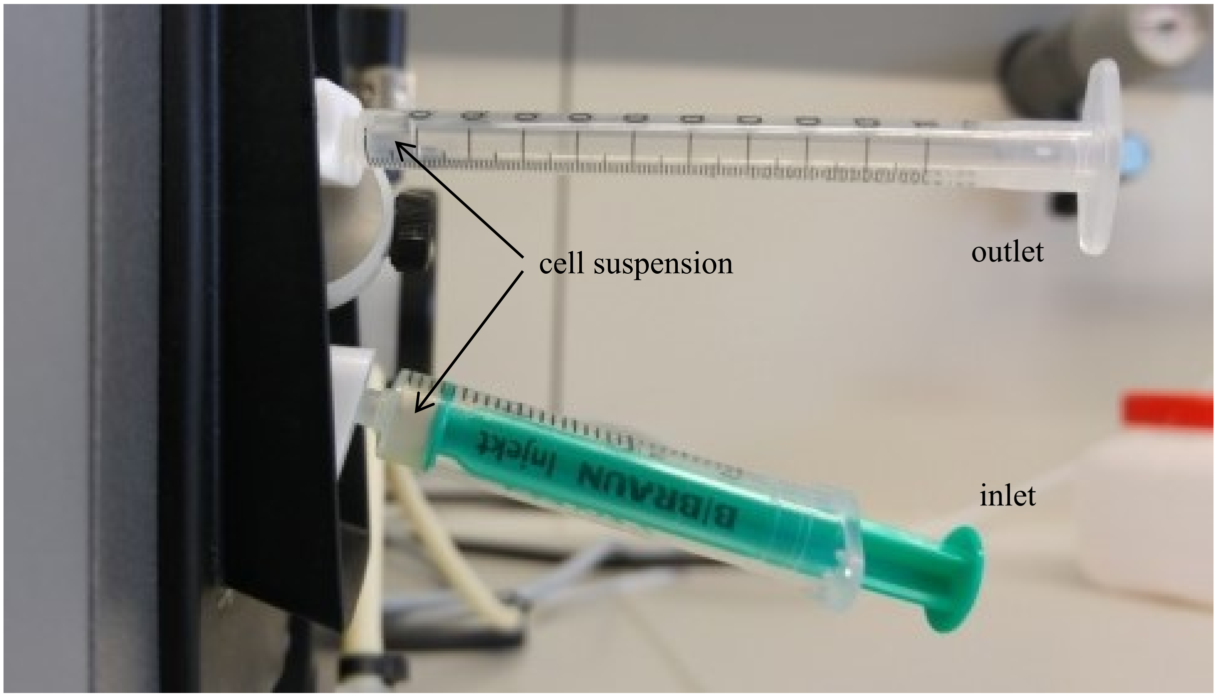

A

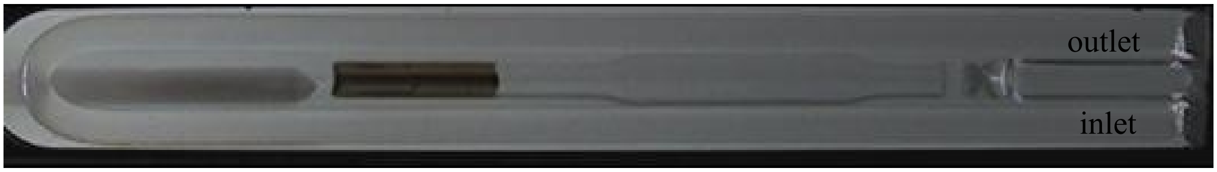

B

Fig. S1. DSA 5000 M density meter test setup (A) 2.0 mL syringe at inlet; about 0.5 mL of originally 2.5 mL cell suspension remained in the syringe while already about 0.1 mL were in the capillary (1.0 mL syringe barrel) at the outlet. (B) The absence of air bubbles in the U-shaped borosilicate glass tube was confirmed with the live camera view of the density meter, Related to Figure 3.

Table S2. Density centrifugation: fluid properties (volumetric mass density, dynamic viscosity, osmolality, pH) of DPBS-LSM mixtures at 100% DPBS, roughly 1.01 to 1.07 g/cm^3 and 100% LSM, Related to Figure 4

Volume fraction of LSM in DPBS [%]	Volumetric mass density [g/cm^3]	Dynamic viscosity [mPa*s]	Osmolality [mOsmol/kg]	pH [ ]
0.00	1.005200	0.998441355	272.5	7.41
6.70	1.009960	1.075552612	273.5	7.39
20.67	1.019560	1.201808165	279.0	7.40
34.64	1.029740	1.531878413	284.0	7.41
48.60	1.039750	1.790569521	294.0	7.41
62.57	1.049710	2.100311091	293.0	7.42
76.54	1.059630	2.592963893	296.5	7.42
90.50	1.069870	3.143607711	285.5	7.39
100.00	1.076800	3.501611099	284.0	7.37
